# Synthesis and Photocatalytic Properties of Four Coordination Compounds Constructed from Two Benzimidazole-Based Asymmetric Polyazocyclic Ligands

**DOI:** 10.3390/molecules28093841

**Published:** 2023-05-01

**Authors:** Chenfei Ren, Jian Li, Xingxing Zhang, Yunyin Niu

**Affiliations:** 1 Green Catalysis Center, and College of Chemistry, Zhengzhou University, Zhengzhou 450001, China; 2 College of Ecology and Environment, Zhengzhou University, Zhengzhou 450001, China; 202012392015688@gs.zzu.edu.cn

**Keywords:** coordination polymer, photocatalytic degradation, antibiotic, water treatment and recycling

## Abstract

In this paper, two benzimidazole derivative ligands were obtained using *o*-phenylenediamine and n-pyridine formaldehyde (n = 3, 4) by amine–aldol condensation reactions, which were reacted with selected inorganic metal salts by ambient temperature volatilization method to give compounds **1–4**: {[(L1)_6_]·[Cu_8_I_8_]} **(1)**, {[L1]·[CuBr]·H_2_O} **(2)**, {[L2]·[CuBr]}_n_ **(3)**, and {[(L2)_4_]·[Cu_4_I_4_]} **(4)**. They were characterized by IR, UV-Vis absorption spectroscopy, thermogravimetric analysis, and single crystal X-ray analysis. Simultaneously, compounds **1–4** were found to possess photocatalytic degradation of ciprofloxacin (CIP) by preliminary experimental investigations.

## 1. Introduction

**Figure 1:**
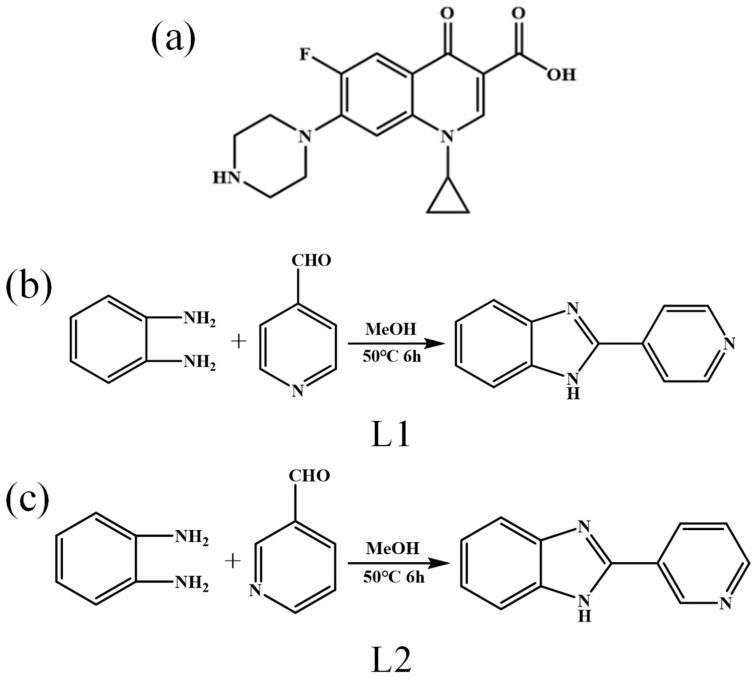
(**a**) Chemical formula for CIP. (**b**) Chemical reaction equation of ligand L1. (**c**) Chemical reaction equation of ligand L2.

In recent years, the presence of pharmaceutical residues in wastewater and their detrimental effects on biological ecosystems has attracted worldwide attention. Drug residues are discharged into the aquatic environment through various channels, such as the pharmaceutical industry, hospital wastewater, and human and livestock excrement, causing pollution to the living environment. Antibiotics are an important part of human and veterinary medicine [1]. Residues of antibiotics account for a large part of pharmaceutical contamination due to the high rate of consumption of antibiotics not only in humans but also in aquaculture and livestock medicines [2]. Ciprofloxacin (CIP), as a typical third-generation fluoroquinolone antibiotic, is difficult to remove by conventional treatment techniques due to its chemical stability and non-biodegradability (Figure 1a). Therefore, there is an urgent need to develop effective technologies to degrade antibiotics in wastewater [3]. Unfortunately, traditional chemical and physical methods are inefficient for its removal, and new biochemical treatments often produce more toxic by-products. At present, the migration behavior and degradation mode of antibiotics in water environment have become a hot research topic. In recent years, photocatalysis technology has been widely used in water pollution control, which has important application prospect and potential [4,5,6,7]. The photocatalytic degradation process provides an ideal way for the governance and degradation of CIP [8,9,10]. Compared with other methods mentioned above, the use of photocatalytic technology can effectively degrade CIP without secondary pollution.

In recent years, coordination compounds as an emerging material for photocatalysis have been widely used in water treatment and recycling fields [11,12,13,14,15]. The benzimidazole ligands are usually used as N-donor ligands, which can adopt different conformations according to the coordination geometry requirements of different metal ions, and serve as donors and acceptors of hydrogen bonds [16,17,18,19,20,21]. This type of ligand has three aromatic rings (pyridine ring, benzene ring, and imidazole ring), which is easy to form a conjugated system that is conducive to strengthening the overall structure; and there are three nitrogen atoms in this type of molecule, they can be obtained by using nitrogen atoms to participate in the coordination with metal ions, while other nitrogen atoms easily form hydrogen-bonding interactions, which further extend the structure to a three-dimensional supramolecular network. Based on this, in this paper, two benzimidazole ligands L1 and L2 (Figure 1b,c) [21] were selected, synthesized, and assembled with metal salts to obtain compounds **1–4** through room temperature reaction, and the obtained compounds were quantitatively produced and their photocatalytic degradation ability to ciprofloxacin containing wastewater was investigated. In addition, the effects of coexisting anions and organic compounds on the removal of CIP were also discussed.

## 2. Results

### 2.1. Synthesis of Compounds

**Scheme 1:**
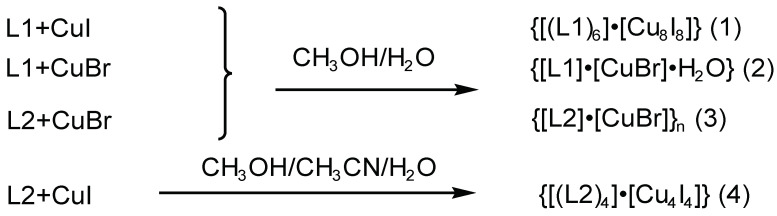
The syntheses of compounds **1–4**.

Over the years, our laboratory has accumulated some experience in the synthesis of single crystals of complexes. The synthesis of the compounds in this paper fully refers to the previous experience of our research group. Two ligands of benzimidazoles were obtained through the condensation reaction of *o*-phenylenediamine and n-pyridine formaldehyde through amine–aldehyde condensation. Specific inorganic metal salts were reacted to obtain compounds **1–4** (Scheme 1).

### 2.2. Description of Crystal Structure

#### 2.2.1. Crystal Structure of Compound **1**

**Figure 2:**
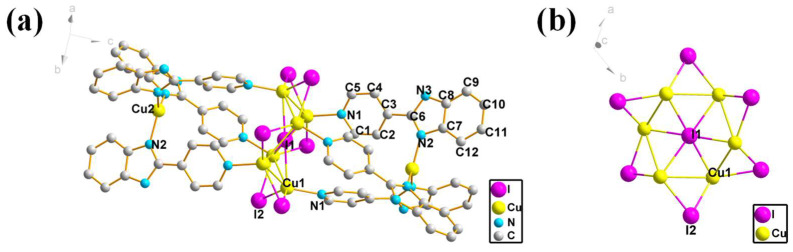
(**a**) Structural monomer diagram of compound **1**. (**b**) Cu-I polygonal structure formed in compound **1**. (The H atom is hidden.)

Compound **1** was obtained by evaporation at room temperature, and its crystal form was red block. X-ray single crystal diffraction analysis showed that compound **1** belonged to a trigonal system, space group R-3. It can be seen from Figure 2 that L1 is a bidentate ligand composed of benzimidazole and pyridine rings. The crystal structure of the complex is shown in Figure 2, and its basic structural unit contains six L1 bidentate ligands; and [Cu_8_I_8_] coordinated with it. As shown in Figure 2a, compound **1** is a multinuclear complex. In compound **1**, both the nitrogen atom of the pyridine ring and the nitrogen atom of the imidazole ring on the bidentate ligand L1 are involved in the coordination with monovalent copper. The nitrogen atoms on the pyridine ring of each of the six ligands L1 are coordinated to six monovalent copper atoms, which are all coordinated to one N atom and three iodines [Cu1-I1 = 2.7229(10) Å, Cu1-I2 = 2.6728(13) Å, Cu1-N1 = 2.050(6) Å]. The remaining two monovalent copper atoms are in a three-coordination mode, which are, respectively, coordinated with the nitrogen atoms of the imidazole ring on the three bidentate ligands L1[Cu2-N2 = 1.979(5) Å]. Through the coordination of monovalent copper with the nitrogen atom on the pyridine ring, a six-pointed star polygonal structure [Cu_6_I_7_] composed of Cu-I is formed in the middle of compound **1**, as shown in Figure 2b. It should be noted here that the structure of the hexagram cluster is the same as that of the literature [22], so we boldly conclude that the two asymmetric ligands have similar template effects. In addition, it can be seen from correlation calculations that the pyridine rings are approximately coplanar, and the dihedral angle between the plane N(1)C(1)C(2)C(3) and the plane N(1)C(5)C(4)C(3) is 2.123(334)°. Benzimidazole is also approximately coplanar, where the dihedral angle between plane C(6)N(3)C(8)C(7)N(2) and plane C(9)C(8)C(7)C(12)C(11)C(10) is 1.290(245)°. The length of the Cu-N bond ranges from 1.979(5) to 2.476(9) Å; the length of the Cu-I bond ranges from 2.439(10) to 2.7229(10) Å. According to the analysis, the dihedral angle of pyridine ring and benzimidazole is 22.841(189)°.

#### 2.2.2. Crystal Structure of Compound **2**

**Figure 3:**
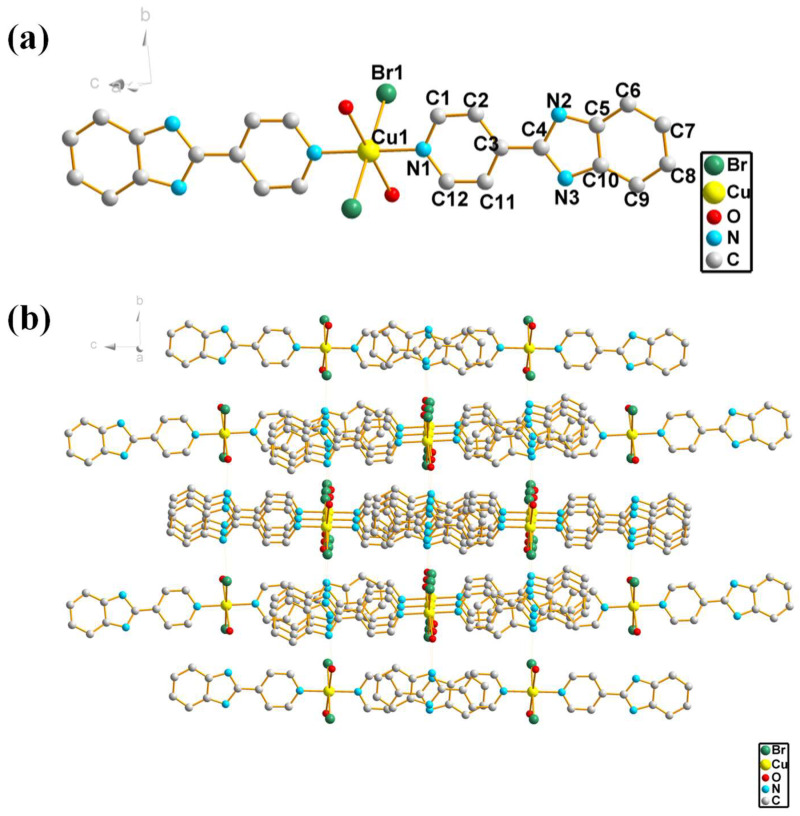
(**a**) The structural monomer diagram of compound **2**. (**b**) The crystal packing diagram of compound **2** in the a-direction. (The H atom is hidden.).

Compound **2** is obtained by evaporation at room temperature and its crystal form is green block. It must be mentioned that the valence state of Cu atoms in compound 2 has changed from +1 (CuBr) to +2 (C_24_H_22_Br_2_CuN_6_O_2_) during the self-assembly process at room temperature. X-ray single crystal diffraction analysis shows that compound **2** belongs to monoclinic system and space group P21/c. The unit cell parameters of compound **2** are as follows: a = 7.1021(13) Å; b = 11.769(2) Å; c = 14.147(3) Å; α = 90°; β = 99.243(7)°; γ = 90°. It can be seen from Figure 3a that L1 is a monodentate ligand, and Figure 3a below is a monomer diagram of compound **2**, which shows that compound **2** is a mononuclear complex. Its smallest structural unit contains an L1 ligand, a copper atom, a bromine atom, and a water molecule. Among them, the copper atom forms a mononuclear complex by coordinating with the nitrogen atom on the pyridine ring. The associated bond lengths are Cu1-Br1 = 2.5170(5) Å, Cu1-N1 = 1.998(3) Å. The structure of compound 8 in the literature [21] is similar to that of compound 2 in this paper, except for metal salt. The two compounds have different hydrogen bond strengths due to different atoms that form hydrogen bonds. Figure 3b shows the crystal packing diagram of compound **2** in the a-direction, and it can be concluded that compound **2** is a 0D structure. In addition, the relevant calculations show that the pyridine ring is approximately coplanar, where the dihedral angle between the plane N(1)C(1)C(2)C(3) and the plane N(1)C(12)C(11)C(3) is 0.240(177)°; the benzimidazole is also approximately coplanar, where the dihedral angle between the plane C(4)N(2)C(5)C(10)N(3) and the plane C(5)C(6)C(7)C(8)C(9)C(10) is 0.845(116)°. According to the analysis, the dihedral angle between the pyridine ring and benzimidazole is 2.357(85)°, which means that the ligand L1 is approximately coplanar in this structure.

#### 2.2.3. Crystal Structure of Compound **3**

**Figure 4:**
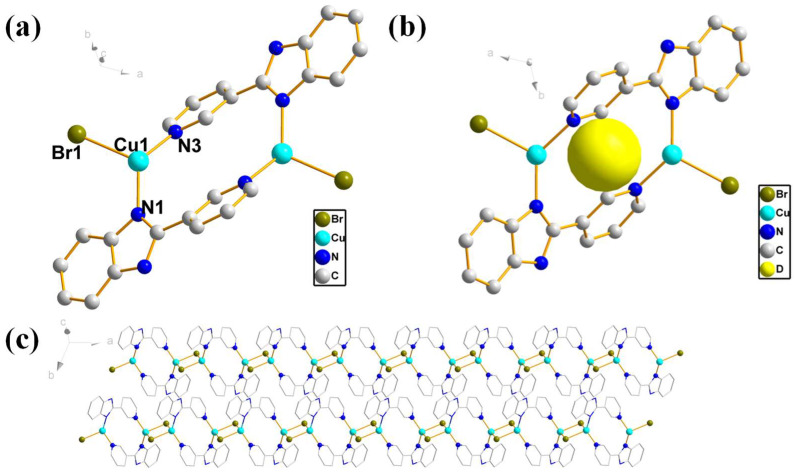
(**a**) Monomer structure diagram of compound **3**. (**b**) Pseudoatoms added to the holes of compound **3**. (**c**) Layered packing diagram of compound **3**. (The H atom is hidden.).

Compound **3** is a 1D structure. X-ray single crystal diffraction analysis showed that compound **3** belonged to triclinic system, space group P-1. The unit cell parameters of compound **3** are a = 7.8416(9) Å, b = 8.6988(9) Å, c = 9.9539(11) Å; α = 109.736(4)°, β = 94.939(4)°, and γ = 113.314(3)°. Figure 4a is the structural monomer diagram of compound **3**. It can be seen from the figure that L2 is a bidentate ligand, and the N atom on the imidazole ring and the N atom on the pyridine ring are involved in the coordination with monovalent copper. The structure of the compound in the literature [23] is basically similar to that of compound **3**, except that the position of the N atom in the pyridine ring in the ligand and the metal salt used are different. The dihedral angle of pyridine ring and benzimidazole ring of the compounds in the literature is 54.6°, while the dihedral angle of the two rings in this paper is 38.357(81)°. In general, when ligand L2 is a monodentate ligand, the two rings are substantially coplanar; while when ligand L2 is a bidentate ligand, the two rings are not coplanar [23]. Moreover, compared to the structure in reference [24], the copper atom of compound 3 in this paper is coordinated with two types of N atoms in the ligand, namely N1 and N3. The structure in reference [24] is that the copper atom is coordinated with N atoms on benzimidazole, forming a one-dimensional chain structure. At the same time, as can be seen from Figure 4b, there is a ring in compound **3** into which we insert a pseudo-atom D. By relevant calculations, we conclude that the macrocyclic ring of compound **3** can pass through a molecule or substance with a radius of 1.41875 Å. The associated bond lengths are Cu1-N1 = 1.983(2) Å,Cu1-N3^2^ = 2.025(2) Å. Figure 4c is the layered packing diagram of compound **3**, and it can also be observed that compound **3** has a 1D chain structure.

#### 2.2.4. Crystal Structure of Compound **4**

**Figure 5:**
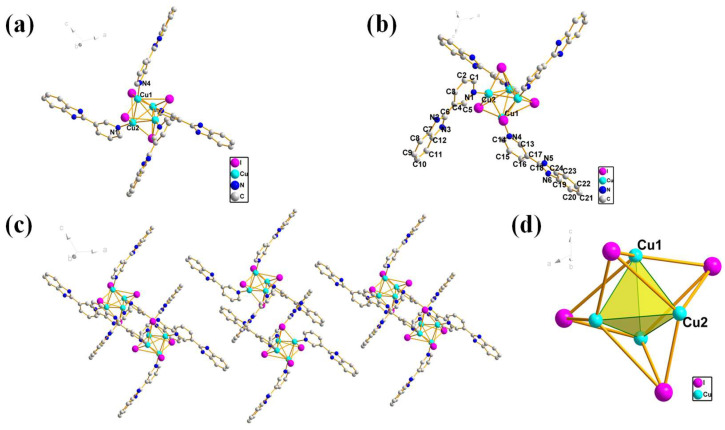
(**a**) The structural monomer diagram of compound **4**. (**b**) The structure diagram of compound **4** with atomic label. (**c**) The unit cell packing diagram of compound **4**. (**d**) Cu-I polygonal structure formed in compound **4**. (The H atom is hidden.).

Compound **4** is a polynuclear cubane-like anion structure. X-ray single crystal diffraction analysis shows that compound **4** belongs to the monoclinic system, C2/c space group. The unit cell parameters of compound **4** are a = 37.424(4) Å, b = 10.1253(11) Å, c = 16.5226(18) Å; α = 90°, β = 115.919(7)°, γ = 90°. Figure 5a is the structural monomer diagram of compound **4**. It can be seen from the figure that: L2 is a monodentate ligand, and the N atom on the pyridine ring of the ligand L2 participates in the coordination with monovalent copper, while the N atom on the imidazole ring is not involved in coordination. The monomer structure of the compound is like a windmill, and the ligand L2 is like a blade on a windmill. The associated bond length are Cu1-N4 = 2.026(4) Å, Cu2-N1 = 2.030(4) Å. In addition, as shown in Figure 5b, according to the analysis, the dihedral angle of the plane where the ligand L1 containing N1 is located, i.e., the plane N(1)C(1)C(2)C(3)C(4)C (6)N(2)C(7)C(8)C(9)C(10)C(11)C(12)N(3), and the plane where the ligand L1 containing N4 is located, i.e., the plane N(4)C(14)C(15)C(16)C(17)N(6)C(19)C(20)C (21)C(22)C(23)C(24)N(5), is 86.055(38)°, approximately perpendicular to each other. Figure 5c is the unit cell packing diagram of compound **4**, like many small windmills stacked together. The structure of compound **4** is similar to that of compound 2 in the literature [25], in which the ligands of both compounds are the same, but the difference is the polygonal structure. Interestingly, the ligand portion of compound **4** resembles a blade, while the polygonal metal salt in the middle of compound 2 in the literature [25] resembles a blade. As can be seen from Figure 5d, the Cu-I polygon contains a tetrahedron about Cu.

### 2.3. Thermogravimetry

**Figure 6:**
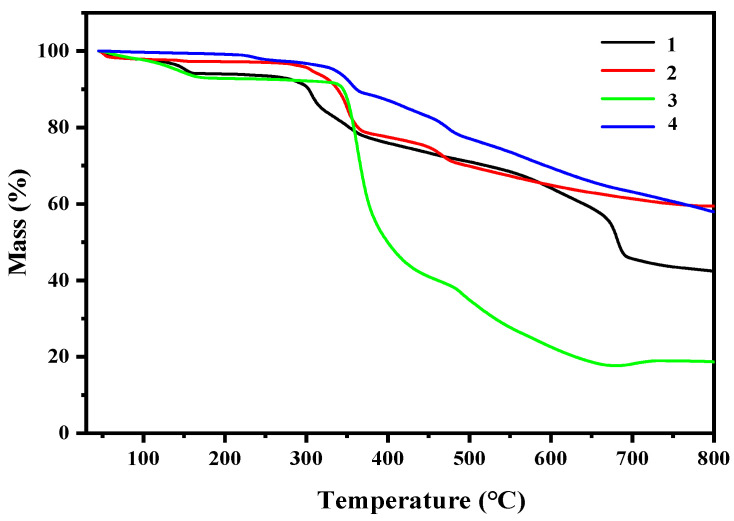
Thermogravimetric curves of compounds **1–4**.

Under N_2_ atmosphere, the four compounds was tested by thermogravimetry at a temperature range of 30–800 °C, and the thermogravimetric curve is shown in Figure 6. By analyzing the thermogravimetric curve, we can obtain information related to the quality of the sample and its possible intermediate products, including their composition, thermal stability, thermal decomposition, and generated products. It can be seen from the figure that these compounds are basically stable when the temperature is lower than 300 °C, and they begin to decompose when the temperature reaches above 300 °C. The weight loss in the range of 30–400 °C may be the pyrolysis of the ligand. When it reaches above 400 °C, it is the decomposition of inorganic metal salt components.

### 2.4. Optical Band Gap of Compounds ***1***, ***2***, ***3***, ***4***

**Figure 7:**
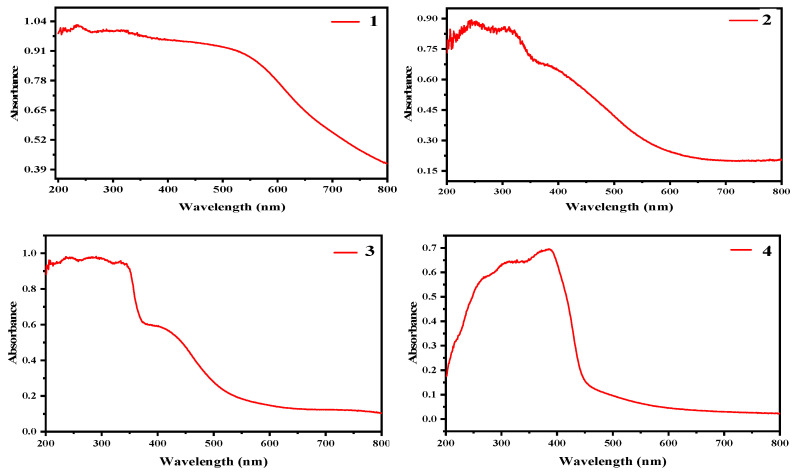
UV-Vis Diffuse Reflection Spectrogram of Compounds **1–4**.

**Figure 8:**
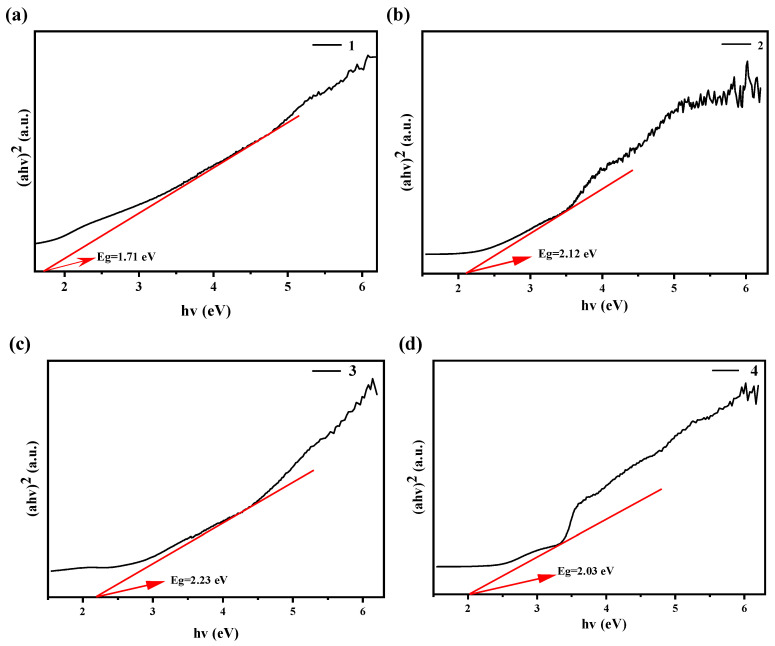
The band gap energy diagrams of compounds **1** (**a**), **2** (**b**), **3** (**c**), and **4** (**d**).

In order to explore the energy band structures and their respective light absorption characteristics of the compounds, they were measured by solid ultraviolet diffuse reflectance spectroscopy (UV-vis-NIRCary5000). The UV-Vis diffuse reflectance spectroscopy of compounds **1–4** were measured using samples of compounds **1–4** at room temperature, the associated spectra are shown in Figure 7. According to the preliminary experiments on the photocatalytic degradation of CIP of compounds **1–4**, it was found that compounds **1–4** had good photodegradation effects on CIP. Therefore, the optical properties of compounds **1–4** were characterized by UV-Vis diffuse reflectance spectroscopy. The photoconductive properties of compounds **1–4** with better degradation performance were studied to determine their respective forbidden band width/band gap energy. Calculate the band gap energy (E_g_) of the compound according to Ahv = C(hv − E_g_)^2^ [26] where A is the absorption coefficient, h is Planck’s constant, v is the incident light frequency, and C is a constant. The variation of (αhv)^2^ as a function of incident photon energy is shown in Figure 8. The band gap energy values E_g_ of compounds **1–4** are 1.71 eV, 2.12 eV, 2.23 eV, and 2.03 eV, respectively. This shows that they are all potential semiconductor materials.

### 2.5. Photocatalytic Properties

**Figure 9:**
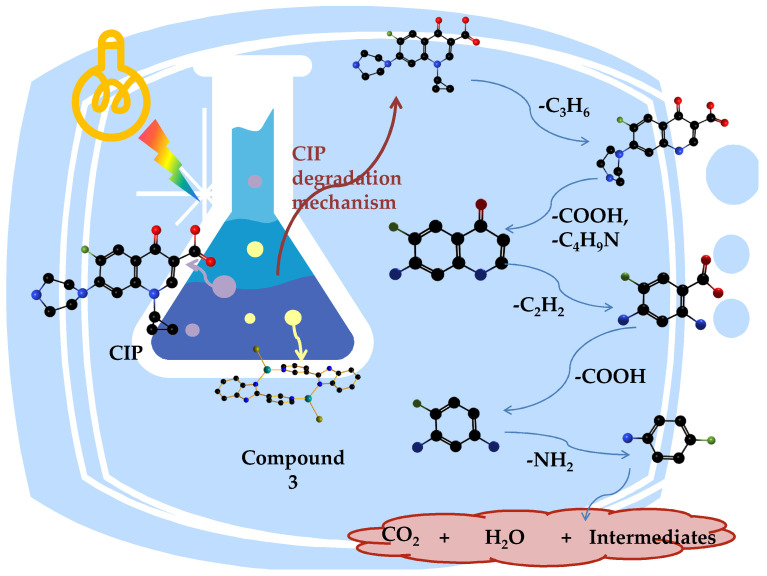
The degradation mechanism of CIP (using compound 3 as an example).

CIP is a typical antibiotic. Here, we will first discuss the degradation mechanism of CIP [27]. It has been reported that the products of CIP degradation are CO_2_, H_2_O, and intermediates, as shown in Figure 9. In order to explore the photodegradation performance of compounds **1–4** as photocatalysts on CIP, photocatalytic degradation experiments were carried out on CIP wastewater under visible light irradiation. In the whole photodegradation reaction, a 500 W mercury lamp was used as the visible light source, tap water was used as the cooling water, and the pH value of the CIP solution was adjusted with 0.1 mol·L^−1^ HCl or NaOH. 10 mg of the catalyst was suspended in CIP (20 mL, 20 mg·L^−1^). To eliminate the adsorption of compounds on CIP, the suspensions were stirred in the dark for 30 min before light irradiation to achieve adsorption-desorption equilibrium. During visible light irradiation, samples were taken at intervals to measure absorbance with a UV-Vis spectrophotometer (UV5500PC).

**Figure 10:**
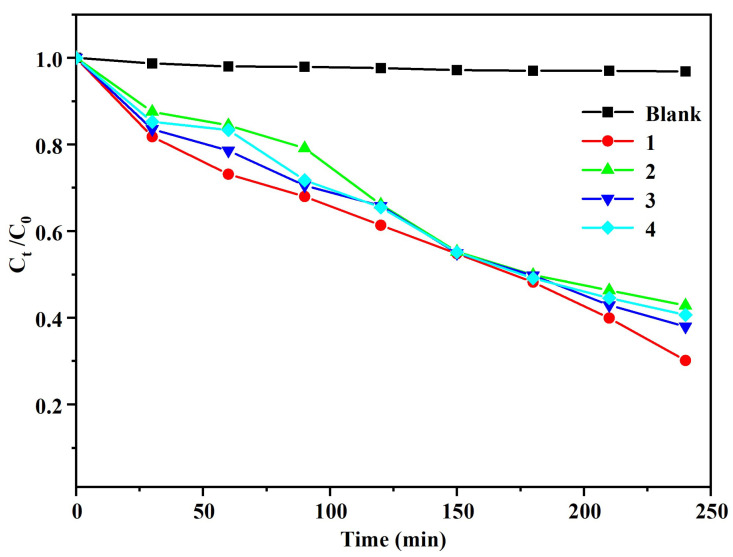
The photocatalytic degradation of CIP by compounds **1–4**.

Figure 10 shows the effect of light time on the photocatalytic degradation of CIP by compounds **1–4**. The degradation effects of compounds **1–4** on CIP are obvious. With the increase in light time, the concentration of CIP gradually decreases, and the degradation efficiencies are 86.95%, 67.18%, 62.02%, and 59.34%. According to the band gap value and photocatalytic degradation efficiency value, we can conclude that the smaller E_g_ is, the higher the photocatalytic efficiency is. Of course, there are many other factors affecting photocatalytic activity, such as separation and capture of photogenerated electrons and holes, crystal structure, lattice defects, etc. Here we only discuss the influence of band gap on it.

Due to the better photocatalytic degradation performance of compounds **1** and **3** compared to compounds **2** and **4**, the following discussion focuses on some of the photocatalytic properties of compounds **1** and **3**.

**Figure 11:**
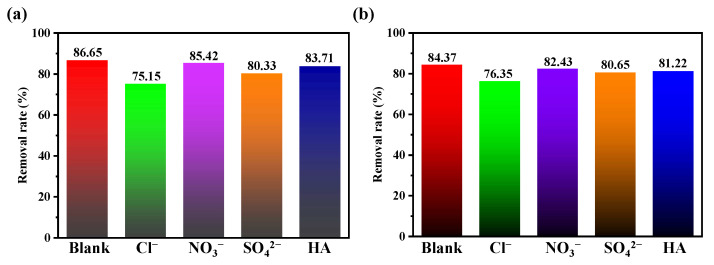
Effect of adding anions and HA on the photodegradation of CIP by compounds **1** (**a**) and **3** (**b**).

Various anions and organic compounds in water can also affect the photocatalytic degradation effect of compounds on antibiotics. Based on this, taking compound **1** and **3** as examples, Cl^−^, NO_3_^−^, SO_4_^2−^, and HA were selected to explore the effects of anions and organic compounds on the photocatalytic degradation of CIP by compounds. The results are shown in Figure 11. When several anions and HA were added to the compound/CIP reaction system, the degradation effect of the compound on CIP was inhibited to varying degrees. HA was selected as the representative of organic compound, which also has a certain impact on the degradation effect of compounds. It can be seen from the figure that Cl^-^ has the most obvious inhibitory effect on the photocatalytic effect. The wastewater with high salt content can be appropriately pretreated during the photocatalytic degradation of CIP in water.

**Figure 12:**
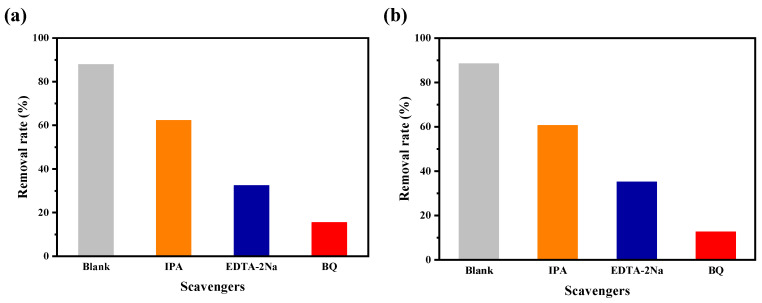
The effect of different radical scavengers on CIP removal by compounds **1** (**a**) and **3** (**b**).

It is generally believed that the main active substances induced by light are photogenerated holes (h^+^), superoxide radical anions (·O_2_^−^), and hydroxyl radicals (·OH), which can act as reaction catalysts in photocatalytic reaction systems. In order to further study the internal mechanism of compounds degrading CIP, and to understand the active radicals that play a major role in the catalytic degradation of CIP, active substance capture experiments were carried out in this paper. Different radical scavengers were added to the photocatalytic reaction system under the same light conditions. Three quenchers used in this experiment: disodium ethylenediaminetetraacetic acid (EDTA-2Na, 1 mmol·L^−1^, h^+^ scavenger), 1 4-benzoquinone (BQ, 1 mmol·L^−1^, ·O_2_^−^ scavenger), and isopropanol (IPA, 1 mmol·L^−1^, ·OH scavenger). It can be seen from Figure 12 that when no capture agent was added, the removal rates of CIP by compounds **1** and **3** were 87.93% and 88.46%, respectively. After adding three capture agents to the above solutions, the photodegradation efficiency of CIP had a certain degree, indicating that ·OH, h^+^ and ·O_2_^−^ are the active substances to degrade CIP. When BQ was added, the inhibitory effect was the most obvious, which indicated that ·O_2_^−^ was the main active substance involved in the photodegradation of CIP catalyzed by the compound, and h^+^ and ·OH played a synergistic effect.

**Figure 13:**
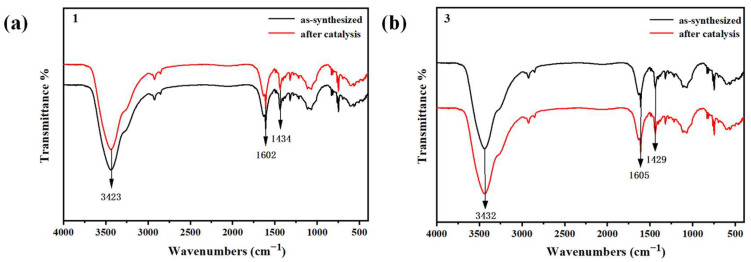
Infrared spectra of compounds **1** (**a**) and **3** (**b**) before and after photocatalytic degradation of CIP.

**Figure 14:**
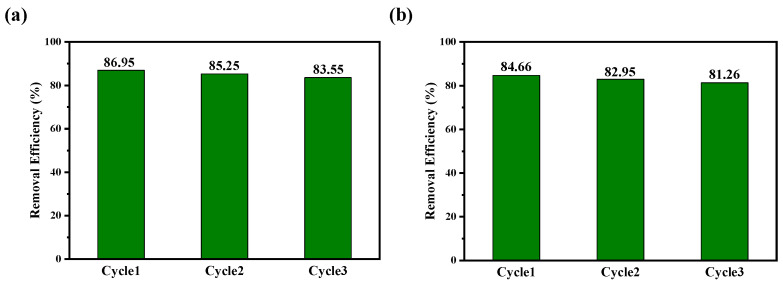
Removal rate of CIP by compounds **1** (**a**) and **3** (**b**) in three cycle experiments.

**Figure 15:**
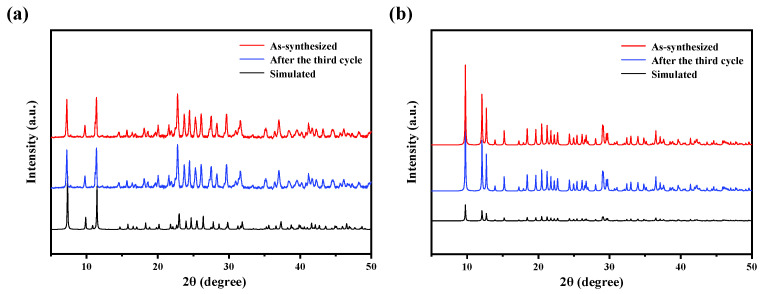
PXRD diagrams of compounds **1** (**a**) and **3** (**b**) before and after three cycle experiments.

In order to investigate the value of the compounds for practical applications of photocatalytic degradation of CIP, the recyclability and stability of the compounds for use as photocatalysts were investigated in this paper. The compound **1** and **3** after the photocatalysis experiment were washed, dried, and recovered, and the mid-far infrared test was carried out on them. The results are shown in Figure 13. It can be seen from the figure that the infrared spectra of the compounds before and after the degradation experiment are basically the same. In addition, around 3423 cm^−1^, there is a N-H stretching vibration peak, and around 1602 cm^−1^ and 1434 cm^−1^, there is a stretching vibration peak of the benzene ring. These characteristic peaks can all prove the presence of benzene ring, N-H bond, and pyridine ring. The photocatalytic stability of compound **1** and **3** is shown in Figure 14. After each photocatalytic degradation of the CIP solution, the collected compound was washed with deionized water several times to wash away the residual CIP on the surface of the compound. The collected compounds were then reintroduced into fresh CIP solution to start a new experiment. In the cycle experiment, all experimental conditions and operations were kept the same as the first experiment. The stability and reusability of compound **1** and **3** in degrading CIP were studied by three consecutive cycle experiments. As shown in Figure 14, the degradation efficiency of CIP did not decrease significantly, and the removal rate of CIP by the compound remained above 80% after three cycles of experiments. The results showed that compound **1** and **3** could be used as a stable photocatalyst for the photocatalytic degradation of CIP. Figure 15 is the powder X-ray diffraction test of compound **1** and **3** after each cycle test, showing that the structure and composition of compound **1** and **3** have not changed before and after the degradation test, and can still maintain its structural integrity.

## 3. Materials and Methods

### 3.1. Materials

The reagents used in the experiment were all commercially analytically pure and were not purified before the experiment. According to the literature and adjusting the synthesis scheme, the ligand 2-Pyridin-n-yl-1H-benzoimidazole (*n* = 3, 4) was prepared by direct reaction of *o*-phenylenediamine and n-pyridine formaldehyde (*n* = 3, 4). Herein, when n is 4, it is called L1, and when n is 3, it is called L2. L1 and L2 belong to Schiff bases. Schiff bases are a class of organic compounds that contain imine or imine characteristic groups (-RC=N-), in which the imine bond (-C=N-) has a solitary pair of electrons in N atom, and can cooperate with various substances. They have the advantages of simple synthesis, easy modification, stability, and flexibility [28].

**Figure 16:**
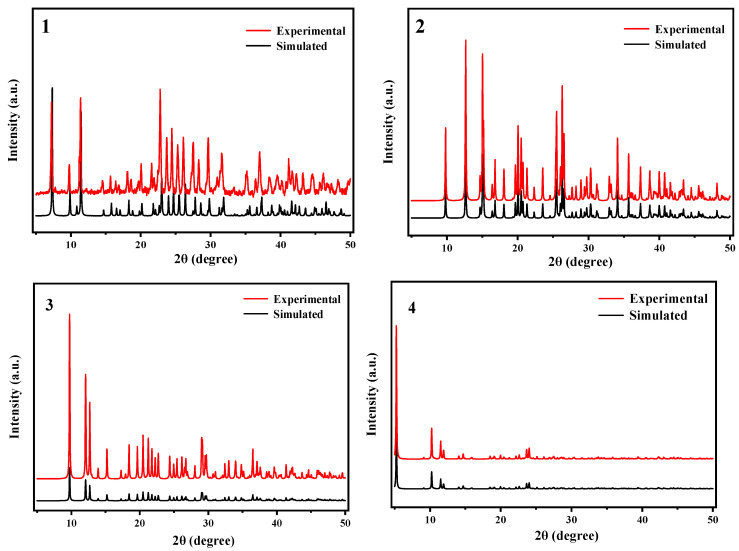
PXRD diagram of compounds **1–4**.

**Figure 17:**
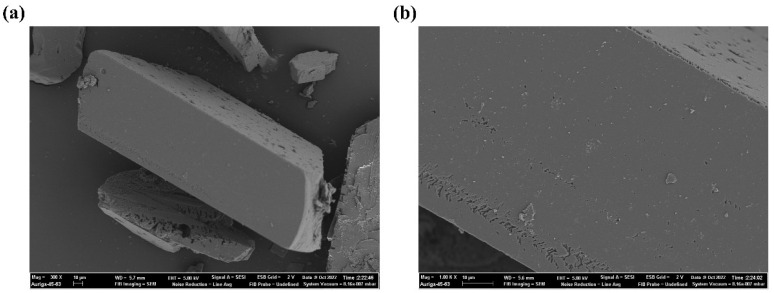
(**a**) Scanning electron micrograph of compound **3**. (**b**) Scanning electron microscopy of compound **3** after magnification.

**Figure 18:**
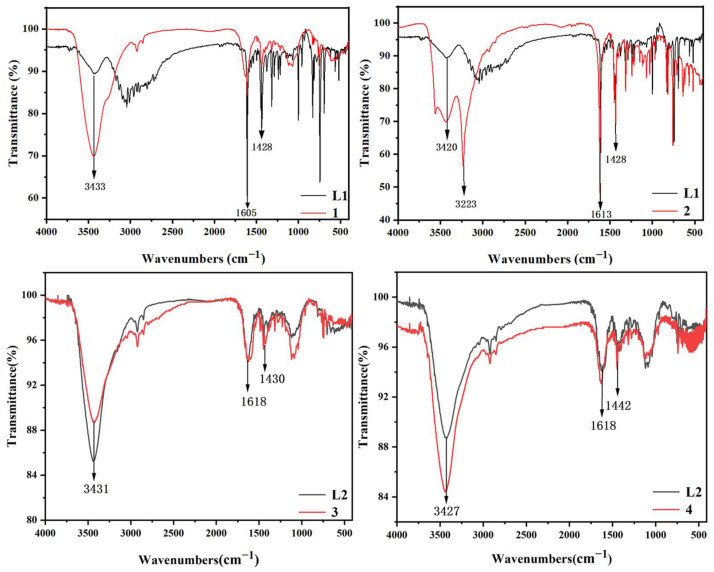
IR Spectrogram of Compounds **1–4**.

X-ray single crystal diffraction: The instrument model is Bruker D8 VENTURE, the manufacturer is Bruker, Karlsruhe, Germany. After the single crystal is cultured, a single crystal with good quality is selected under the microscope as the sample to be tested. Good quality single crystal samples are generally angular, with a smooth surface free of cracks and free of microscopic grains and powdered impurities. OlEX-2 was used to analyze and refine the crystal data collected. The main crystallographic data parameters of the compounds are shown in Table 1. The bond lengths and angles selected for compounds **1–4** are shown in Table 2. The PXRD patterns of compounds **1–4** are shown in Figure 16. It can be seen from the Figure 16 that the experimental data of compounds **1–4** and the simulation data of single crystal have a high degree of fitting, and the comparison patterns are basically the same, indicating that the sample is of high purity, which can be used for subsequent experimental exploration. Simultaneously, we also conducted a scanning electron microscopy of compound **3**. By using scanning electron microscopy, crystal defects can be directly studied. From Figure 17, it can be seen from the electron microscopy that compound **3** may have a porous structure inside and a relatively large specific surface area, so we conclude that its photocatalytic performance may be good. Infrared spectroscopy (IR): Infrared spectra were measured in the wave number range of 400~4000 cm^−1^ by using a potassium bromide press using an instrument model Bruker VECTOR27, Karlsruhe, Germany, as shown in Figure 18. In addition, around 3430 cm^−1^, there is a N-H stretching vibration peak, and around 1610 cm^−1^, there is a stretching vibration peak of the benzene ring. These characteristic peaks can all prove the presence of benzene ring, N-H bond, and pyridine ring. In the IR Spectrogram, we made a comparison between the ligand and the compound. Through comparison, we found that the ligand structure did not change after the formation of the complex, and the original characteristic peaks belonging to the ligand did not disappear. Solid UV diffuse reflectance spectroscopy: the instrument model is UV-vis-NIRCary5000, Guangdong, China, barium sulfate is used as a blank control at room temperature, and the scanning range is 200–800 nm. Thermogravimetric analysis (TG): The instrument model is NETZSCHTG209. Take 15 mg of sample and raise the temperature from room temperature to 800 °C under N_2_ atmosphere, and record the mass change of the sample during the heating process. Elemental analysis (EA): The instrument model is Perkin-Elmer 240, Waltham, Massachusetts, USA, and the content of C, H, and N elements in the obtained compound is determined at room temperature.

### 3.2. Photocatalytic Determination

In this paper, the photocatalytic performance of the obtained compound as a photocatalyst was evaluated by studying the degradation effect of the obtained compound on CIP under visible light irradiation. The CIP degradation experiment was completed in a photochemical reaction instrument (HANUO-IV) produced by Shanghai Hanuo Instrument Co., Ltd., Shanghai, China, and a mercury lamp with a wavelength range of 250–720 nm was selected to simulate a visible light source. Before using the instrument, put the magnetic stirrer into the dark box of the main machine, fix the quartz cold trap, put the magnet in the reaction vessel, and then connect the mercury lamp, the reactor, and the cooling water circulation device. During the experiment, 10 mg of the compound was first weighed and added to the reaction vessel, and then the antibiotic solution prepared in advance was added. In order to eliminate the adsorption of compounds on CIP, the reaction solution was stirred in the dark for 30 min before light to achieve adsorption-desorption equilibrium. When the light source is turned on, the low-temperature coolant circulation pump is started to control the internal temperature of the reactor at 25 °C. A 0.22 μm filter was used for each sample taken with a syringe. The absorbance of the degradation product at the maximum absorption wavelength was measured on an ultraviolet-visible spectrophotometer (UV5500PC).

### 3.3. Synthesis of Compounds

#### 3.3.1. Synthesis of {[(L1)_6_]·[Cu_8_I_8_]} (**1**)

L1 (0.0020 g, 0.01 mmol), CuI (0.0019 g, 0.01 mmol) were weighed in two clean penicillin vials, L1 was fully dissolved with 3 mL methanol, 2 drops of water was first added to the vial in which CuI was placed, then an appropriate amount of KI was added and sonicated until CuI was completely dissolved, then 3 mL methanol was added. The methanol solution of L1 was added drop by drop to the vial in which the methanol solution of CuI was placed, stirred on a magnetic stirrer for 10 min after adding the magnet, filtered the solution in the vial into another clean penicillin vial, labeled and sealed with cling film, pierce 3–4 small holes, volatilized the solvent slowly at room temperature, recorded the time of dispensing the vial and observed regularly, about 4 days later there were red lumpy crystals at the bottom of the vial. After about 4 days, red lumpy crystals were analyzed at the bottom of the vial. The crystals were collected by filtration, washing and drying, and the yield was about 24%. Mass: 0.0011g. IR (KBr, cm^−1^): 3440 (s), 2924 (w), 1610 (m), 1437 (w), 1321 (w), 1069 (w), 831 (w), 816 (w), 764 (w), 748 (w), 566 (w).Anal. Calcd for C_72_H_54_Cu_8_I_8_N_18_ (2694.85): C, 32.06; H, 2.00; N, 9.35%. Found: C, 32.11; H, 2.01; N, 9.42%.

#### 3.3.2. Synthesis of {[L1]·[CuBr]·H_2_O} (**2**)

The synthesis of {[L1]·[CuBr]·H_2_O} **(2)** is the same as that of {[(L1)_6_]·[Cu_8_I_8_]} **(1)**, except that CuI is replaced by CuBr and KI is replaced by KBr. Green crystals are precipitated at the bottom of the vial after about two weeks. The crystals were filtered, washed, dried, and collected with a yield of about 10%. Mass: 0.0008 g. IR (KBr, cm^−1^): 3561 (m), 3439 (m), 3231 (m), 1618 (m), 1449 (m), 1433 (m), 1404 (w), 1315 (m), 1284 (w), 1241 (m), 1216 (m), 1148 (w), 1113 (m), 1070 (m), 1030 (m), 968 (w).Anal. Calcd for C_24_H_22_Br_2_CuN_6_O_2_ (649.83): C, 44.32; H, 3.39; N, 12.93%. Found: C, 44.39; H, 3.41; N, 12.89%.

#### 3.3.3. Synthesis of {[L2]·[CuBr]}_n_ (**3**)

L2 (0.0040 g, 0.02 mmol) and CuBr (0.0028 g, 0.02 mmol) were weighed into two clean penicillin vials, and L2 was fully dissolved with 4 mL methanol. A total of 2 drops of water were added into the CuBr vial, and then excessive KBr was added. After the CuBr was dissolved, 4mL methanol was added. Add the methanol solution of L2 to the methanol solution of CuBr, add the magneon, stir on the magnetic agitator, filter the solution in the vial into another clean penicillin vial, label it and seal it with plastic wrap, pierce 3–4 holes, slowly evaporate the solvent at room temperature, record the vial preparation time and observe regularly. After about two days, yellow mass crystals formed at the bottom of the bottle. Filtration, washing, dries collection; the yield of crystal is about 78%. Mass: 0.0031 g. IR (KBr, cm^−1^): 3839 (w), 3440 (m), 2923 (w), 1635 (w), 1558 (w), 1447 (w), 1317 (w), 1120 (w), 814 (w), 705 (w), 619 (w), 440 (w).Anal. Calcd for C_12_H_9_BrCuN_3_ (338.48): C, 42.54; H, 2.66; N, 12.40%. Found: C, 42.51; H, 2.69; N, 12.38%.

#### 3.3.4. Synthesis of {[(L2)_4_]·[Cu_4_I_4_]} (**4**)

L2 (0.0040 g, 0.02 mmol) and CuI (0.0019 g, 0.01 mmol) were weighed into two clean penicillin vials, respectively. L2 was fully dissolved with 4 mL methanol, and then 2 drops of water were added into the vial placed with CuI and appropriate amount of KI was added. After it was completely dissolved, 4 mL acetonitrile was added. Add the methanol solution of L2 drop by drop to CuI’s acetonitrile solution, add the magneton and stir it on the magnetic agitator for 10 min, filter the solution in the vial into another clean penicillin vial, label it and seal it with plastic wrap, pierce 3–4 holes, slowly evaporate the solvent at room temperature, record the vial preparation time and make regular observation. After about a week, thin yellow crystals formed at the bottom of the bottle. Filtration, washing, dries collection, the yield of crystal is about 78%. Mass: 0.0015 g. IR (KBr, cm^−1^): 3758 (w), 3445 (m), 2922 (w), 1635 (w), 1558 (w), 1447 (w), 1319 (w), 1281 (w), 1114 (w), 973 (w), 779 (w), 659 (w), 450 (w). Anal. Calcd for C_48_H_36_Cu_4_I_4_N_12_ (1541.78): C, 37.36; H, 2.33; N, 10.90%. Found: C, 37.31; H, 2.35; N, 10.89%.

## 4. Conclusions

Herein, the ligands L1 and L2 were synthesized, and then four complexes were obtained by the reaction of these two ligands with selected inorganic metal salts. The preparation of compounds **1–4** was demonstrated by single crystal X-ray diffraction, infrared, and PXRD characterization. TGA test showed that the compounds had high thermal stability. Simultaneously, compounds **1–4** showed photocatalytic degradation of CIP. The values of the compounds **1** and **3** as photocatalyst were proved by cyclic experiments. ·O_2_^−^ is the main active substance in CIP degradation process. Finally, we can conclude that the smaller E_g_ is, the higher the photocatalytic efficiency is. Therefore, compounds 1 and 3 can be applied to the wastewater treatment in the future. The photocatalytic degradation experiments under different light sources would also be increased for the control experiment in the future.

## Figures and Tables

**Table 1:** Crystal data and structural refinement details of complexes **1–4**.

Compounds	1	2	3	4
Empirical formula	C_72_H_54_Cu_8_I_8_N_18_	C_24_H_22_Br_2_CuN_6_O_2_	C_12_H_9_BrCuN_3_	C_48_H_36_Cu_4_I_4_N_12_
Formula weight	2694.85	649.83	338.67	1542.65
Temperature/K	100.2(4)	298.00	300.00	297.0
Crystal system	trigonal	monoclinic	triclinic	monoclinic
Space group	R-3	P21/c	P-1	C2/c
a/Å	16.2957(3)	7.1021(13)	7.8416(9)	37.424(4)
b/Å	16.2957(3)	11.769(2)	8.6988(9)	10.1253(11)
c/Å	23.1449(5)	14.147(3)	9.9539(11)	16.5226(18)
α/°	90	90	109.736(4)	90
β/°	90	99.243(7)	94.939(4)	115.919(7)
γ/°	120	90	113.314(3)	90
Volume/Å^3^	5322.7(2)	1167.1(4)	567.63(11)	5631.2(11)
Z	3	2	2	4
ρcalcg/cm^3^	2.522	1.849	1.981	1.820
μ/mm^−1^	5.890	4.396	5.420	3.726
F(000)	3804.0	646.0	332.0	2944.0
Crystal size/mm^3^	0.12 × 0.11 × 0.1	0.35 × 0.23 × 0.14	0.27 × 0.23 × 0.22	0.25 × 0.23 × 0.17
Reflections collected	13,736	27,464	12,706	71,651
Independent reflections/R_int_/R_sigma_	2760, 0.0286, 0.0202	2813, 0.1060, 0.0520	2613, 0.0516, 0.0352	6401, 0.1346, 0.0616
Data/restraints/parameters	2760/0/178	2813/0/163	2613/0/154	6401/0/307
Goodness-of-fit on F^2^	1.128	1.006	1.015	0.987
Final R indexes [I > =2σ (I)]	R_1_ = 0.0393, wR_2_ = 0.1054	R_1_ = 0.0364, wR_2_ = 0.0859	R_1_ = 0.0286, wR_2_ = 0.0826	R_1_ = 0.0397, wR_2_ = 0.0819
Final R indexes [all data]	R_1_ = 0.0412, wR_2_ = 0.1063	R_1_ = 0.0706, wR_2_ = 0.0970	R_1_ = 0.0339, wR_2_ = 0.0856	R_1_ = 0.0852, wR_2_ = 0.0929
Largest diff. peak/hole/e Å^−3^	3.22/−3.03	0.59/−0.58	0.63/−0.56	0.66/−0.46

**Table 2:** The main bond length and bond angle of compounds **1–4**.

Compound **1**					
I2-Cu1^1^	2.5916 (12)	I2-Cu1	2.6728 (13)	I1-Cu1^2^	2.7229 (10)
I1-Cu1^3^	2.7229 (10)	I1-Cu1^4^	2.7229 (10)	I1-Cu1^1^	2.7229 (10)
I1-Cu1	2.7229 (10)	I1-Cu1^5^	2.7229 (10)	Cu1-Cu1^5^	2.9812 (13)
Cu1-Cu1^1^	2.9812 (13)	Cu1-N1	2.050 (6)	Cu2-N2^3^	1.979 (5)
Cu2-N2	1.979 (5)	Cu2-N2^4^	1.979 (5)	N3-Cu2A^6^	2.230 (9)
N1-Cu2A	2.476 (9)	Cu2A-I2A	2.439 (10)	Cu1^1^-I2-Cu1	68.96 (5)
Cu1^1^-I1-Cu1^2^	113.617 (15)	Cu1^3^-I1-Cu1^1^	180.0	Cu1^3^-I1-Cu1^2^	66.384 (15)
Cu1^4^-I1-Cu1^1^	66.384 (15)	Cu1^3^-I1-Cu1	113.619 (15)	Cu1^4^-I1-Cu1^2^	180.0
Cu1^5^-I1-Cu1^4^	66.382 (15)	Cu1^5^-I1-Cu1^1^	113.617 (15)	Cu1^5^-I1-Cu1	180.00 (3)
Cu1^1^-I1-Cu1	66.383 (15)	Cu1^5^-I1-Cu1^3^	66.381 (15)	Cu1^5^-I1-Cu1^2^	113.617 (15)
Cu1^2^-I1-Cu1	66.383 (15)	Cu1^4^-I1-Cu1^3^	113.616 (15)	Cu1^4^-I1-Cu1	113.619 (15)
I2^2^-Cu1-I2	120.62 (4)	I2^2^-Cu1-I1	108.36 (4)	I2-Cu1-I1	106.02 (4)
I2^2^-Cu1-Cu1^2^	56.80 (4)	I2^2^-Cu1-Cu1^1^	113.83 (5)	I2-Cu1-Cu1^1^	54.23 (3)
I2-Cu1-Cu1^2^	152.22 (5)	I1-Cu1-Cu1^1^	56.808 (7)	I1-Cu1-Cu1^2^	56.808 (8)
Cu1^2^-Cu1-Cu1^1^	99.69 (4)	N1-Cu1-I2^2^	111.50 (16)	N1-Cu1-I2	105.02 (16)
N1-Cu1-I1	104.00 (17)	N1-Cu1-Cu1^2^	100.69 (16)	N1-Cu1-Cu11	134.40 (16)
N2^3^-Cu2-N2	119.917 (18)	N2^3^-Cu2-N2^4^	119.916 (18)	N2^4^-Cu2-N2	119.916 (16)
C6-N3-Cu2A^6^	104.0 (4)	C8-N3-Cu2A^6^	123.5 (4)	C6-N2-Cu2	134.8 (4)
C7-N2-Cu2	119.4 (4)	C1-N1-Cu1	118.9 (5)	C1-N1-Cu2A	118.4 (5)
C5-N1-Cu1	123.6 (4)	C5-N1-Cu2A	110.8 (5)	N3^6^-Cu2A-N1	142.9 (4)
N3^6^-Cu2A-I2A	99.8 (3)	I2A-Cu2A-N1	101.0 (3)	Cu2A-I2A-Cu2A^1^	130.1 (4)
Compound **2**					
Br1-Cu1	2.5170 (5)	Cu1-N1^1^	1.999 (3)	Cu1-N1	1.998 (3)
N1-C1	1.350 (4)	N3-C4	1.315 (4)	C1-C2	1.369 (4)
Br1-Cu1-Br1^1^	179.999 (11)	N1^1^-Cu1-Br1	89.84 (7)	N1-Cu1-Br1^1^	89.84 (7)
N1^1^-Cu1-Br1^1^	90.16 (7)	N1-Cu1-Br1	90.16 (7)	N1-Cu1-N1^1^	180.00 (3)
C12-N1-Cu1	120.6 (2)	C1-N1-Cu1	122.2 (2)		
Compound **3**					
Br1-Cu1	2.5678 (5)	Br1-Cu1^1^	2.6537 (5)	Cu1-N1	1.983 (2)
Cu1-N3^2^	2.025 (2)	Cu1-Br1-Cu1^1^	86.720 (15)	Br1-Cu1-Br1^1^	93.280 (15)
N1-Cu1-Br1^1^	108.83 (6)	N1-Cu1-Br1	115.12 (6)	N1-Cu1-N3^2^	128.14 (8)
N3^2^-Cu1-Br1	102.94 (6)	N3^2^-Cu1-Br1^1^	102.43 (6)	C1-N1-Cu1	125.69 (16)
C7-N1-Cu1	127.56 (17)	C11-N3-Cu1^2^	120.63 (17)	C12-N3-Cu1^2^	121.81 (17)
Compound **4**					
I1-Cu1	2.5714 (7)	I1-Cu2	2.5713 (8)	I2-Cu11	2.5872 (8)
I2-Cu2	2.5814 (8)	Cu1-Cu1^1^	2.7688 (15)	Cu1-Cu2	2.6778 (9)
Cu1-Cu2^1^	2.6408 (9)	Cu1-N4	2.026 (4)	Cu2-Cu2^1^	2.7509 (13)
Cu2-N1	2.030 (4)	Cu2-I1-Cu1	62.76 (2)	Cu2-I1-Cu1^1^	61.45 (2)
I1-Cu1-I2^1^	120.48 (3)	I1-Cu1-Cu1^1^	113.44 (2)	I1-Cu1-Cu2^1^	75.37 (3)
I1-Cu1-Cu2	58.62 (2)	I2^1^-Cu1-Cu1^1^	75.34 (3)	I2^1^-Cu1-Cu2^1^	59.16 (2)
I2^1^-Cu1-Cu2	117.82 (3)	Cu2^1^-Cu1-Cu1^1^	59.29 (2)	Cu2-Cu1-Cu1^1^	57.98 (3)
Cu2^1^-Cu1-Cu2	62.29 (3)	N4-Cu1-I1	115.18 (11)	N4-Cu1-I2^1^	108.15 (10)
N4-Cu1-Cu1^1^	118.52 (11)	N4-Cu1-Cu2^1^	167.23 (11)	N4-Cu1-Cu2	128.67 (10)
I1-Cu2-I2	120.99 (3)	I1-Cu2-Cu1^1^	117.91 (3)	I1-Cu2-Cu1	58.62 (2)
I1-Cu2-Cu2^1^	73.48 (3)	I2-Cu2-Cu1^1^	59.38 (2)	I2-Cu2-Cu1	77.04 (3)
I2-Cu2-Cu2^1^	115.44 (2)	Cu1^1^-Cu2-Cu1	62.74 (3)	Cu1-Cu2-Cu2^1^	58.20 (2)
Cu1^1^-Cu2-Cu2^1^	59.52 (3)	N1-Cu2-I1	108.87 (12)	N1-Cu2-I2	112.66 (11)
N1-Cu2-Cu1	167.48 (12)	N1-Cu2-Cu1^1^	128.55 (12)	N1-Cu2-Cu2^1^	120.24 (11)
C14-N4-Cu1	120.5 (3)	C13-N4-Cu1	121.4 (3)	C5-N1-Cu2	120.2 (3)
C1-N1-Cu2	121.5 (3)				

## Data Availability

All data generated or analyzed during this study are included in this article.

## Supplementary Materials

The complete crystallography data of compounds **1**–**4** can be obtained free of charge from The Cambridge Crystallographic Data centre via www.ccdc.cam.ac.uk/structures, accessed on 11 April 2023, with CCDC numbers of 2132905 (for compound **1**), 2133316(for compound **2**), 2255341(for compound **3**) and 2255340 (for compound **4**), respectively. Other related supramolecular coordination compounds constructed by templated assembly with transition metals in our group see CCDC(623644, 623649-50, 623343-44, 623349, 623351, 623352, 629782, 1839494, 1872124).

## Sample Availability

Samples of the compounds are not available from the authors.
